# High-resolution melting analysis reveals genetic polymorphisms in MicroRNAs confer hepatocellular carcinoma risk in Chinese patients

**DOI:** 10.1186/1471-2407-14-643

**Published:** 2014-08-31

**Authors:** Jia-Hui Qi, Jin Wang, Jinyun Chen, Fan Shen, Jing-Tao Huang, Subrata Sen, Xin Zhou, Song-Mei Liu

**Affiliations:** Center for Gene Diagnosis, Medical Research Center, Zhongnan Hospital of Wuhan University, 169 Donghu Road, Wuhan, Hubei 430071 China; Department of Translational Molecular Pathology, The University of Texas MD Anderson Cancer Center, Houston, Texas 77054 USA; Department of Epidemiology, The University of Texas MD Anderson Cancer Center, Houston, Texas USA

**Keywords:** Hepatocellular carcinoma, MicroRNA, High-resolution melting, Single-nucleotide polymorphisms

## Abstract

**Background:**

Although several single-nucleotide polymorphisms in microRNA (miRNA) genes have been associated with primary hepatocellular carcinoma, published findings regarding this relationship are inconsistent and inconclusive.

**Methods:**

The high-resolution melting (HRM) analysis was used to determine whether the occurrence of the SNPs of miR-146a C > G (rs2910164), miR-196a2 C > T (rs11614913), miR-301b A > G (rs384262), and miR-499 C > T (rs3746444) differs in frequency-matched 314 HCC patients and 407 controls by age and sex.

**Results:**

The groups’ genotype distributions of miR-196a2 C > T and miR-499 C > T differed significantly (P < 0.01), both of them increased the risk of HCC in different dominant genetic models (P < 0.01); compared with individuals carrying one or neither of the unfavorable genotypes, individuals carrying both unfavorable genotypes (CT + CC) had a 3.11-fold higher HCC risk (95% confidence interval (CI), 1.89–5.09; P = 7.18 × 10^−6^). Moreover, the allele frequency of miR-499 C > T was significantly different between the two groups, and the HCC risk of carriers of the C allele was higher than that of carriers of the T allele (odds ratio, 1.53; 95% CI, 1.15-2.03; P = 0.003). Further, we found that the activated partial thromboplastin time (APTT) in HCC patients with miR-196a2 CC genotype was longer than patients with TT genotypes (P < 0.05), and HCC patients with miR-499 C allele had higher serum levels of direct bilirubin, globulin, γ-glutamyltranspeptidase, alkaline phosphatase, and lower serum cholinesterase (P < 0.05).

**Conclusions:**

Our findings suggest that the SNPs in miR-196a2 C > T and miR-499 C > T confer HCC risk and that affect the clinical laboratory characteristics of HCC patients.

**Electronic supplementary material:**

The online version of this article (doi:10.1186/1471-2407-14-643) contains supplementary material, which is available to authorized users.

## Background

Hepatocellular carcinoma (HCC) is the third most common cause of cancer-related mortality worldwide [1]. In the United States, approximately 6,000 new HCC cases are diagnosed each year. HCC is not a chemosensitive tumor, and most HCCs are diagnosed at an advanced stage, which often renders intervention ineffective, thereby leading to a high mortality rate [2]. The main known risk factors for HCC are hepatitis B and hepatitis C infection; other key risk factors, which vary from country to country, include exposure to aflatoxin B1, excessive alcohol consumption, smoking, diabetes, male sex, and genetic factors [3–5].

Previous studies have shown that single-nucleotide polymorphisms (SNPs) in microRNAs (miRNAs) may contribute to tumorigenesis owing to their ability to change the expression, regulation, and/or function of miRNAs [6–9]. miRNAs are a class of small, non-coding RNAs 17–25 nucleotides in length that are conserved across species and can regulate gene expression by binding to complementary sequences in the 3′- untranslated regions of target mRNAs [6, 9]. As oncogenes or tumor suppressor genes, miRNAs play important roles in human cancer progression, affecting tumor invasiveness, metastasis, EMT and other clinical characteristics [9]. Genetic variations in miRNAs are confirmed to relate with renal cell carcinoma [10], non-small cell lung cancer [11], HCC [12–15], digestive system cancer [16], breast cancer [17, 18], gastric cancer [19, 20], colorectal cancer [21, 22], cervical squamous cell carcinoma [23], ovarian cancer [24], papillary thyroid carcinoma [25], adult glioma [26] and oral cancer [27]. However, the exact mechanism by which miRNA expression levels are altered in different cancers remains unknown. Researchers have recently proposed that a large number of potentially functional miRNA-related SNPs are potential cancer biomarkers. Among these, the SNPs in miR-146a C > G, miR-196a2 C > T, and miR-499 C > T, which have been reported to be associated with liver cancer [12–15], breast cancer [17, 18], gastric cancer [19, 20] and colorectal cancer [21, 22]. In particular, the rs11614913 SNP in miR-196a2 [12, 15], the rs2910164 SNP in miR-146a [14, 15] and the rs3746444 SNP in miR-499 [13] are likely associated with HCC risk. miR-499 C > T may play an important role in HCC pathogenesis by regulating ets1, which plays a fundamental role in extracellular matrix degradation, a process required for tumor cell invasion and migration [28]. The rs3746444 SNP in miR-499 C > T has also been associated with susceptibility to hepatitis B virus–related HCC [13]. Guo *et al.* also found the significant association between the SNP in miR-196a2 and increased susceptibility to colorectal cancer and HCC [16]. miR-146a also played key roles in regulating the angiogenic activity of endothelial cells in HCC through BRCA1-PDGFRA pathway and regulating the sensitivity of HCC cells to the cytotoxic effects of IFN-α through SMAD4 [29, 30]. The C > G polymorphism of miR-146 precursor affects the production of mature miR-146a and is associated with the risks of HCC, adult glioma and gastric cancer [14, 20, 26]. miR-301 is an interesting miRNA, which was differentially expressed in HCC compared with adjacent benign liver [31] and was down-regulated in HCV-infected Huh7.5 cells and subsequently up-regulated following interferon-α treatment [32].

However, the meta-analysis revealed that the miR-146a C > G (rs2910164) variant was associated with a decreased HCC risk among Asian and male populations and no significant association was observed between the SNP and risk for HCC in the female populations [15]. They have not found a linkage between miRNA-related SNPs and HCC, such as no significant association between the SNP of miR-146a C > G and HCC risk [13, 33], no significant correlation between the miR-499 rs3746444 polymorphism and HCC risk [15, 33], and no significant association between the miR-196a2 SNP and the risk of hepatitis B virus–related HCC [12]. Even if HCC risk was significantly lower in male patients with the miR-196a2 TT genotype or T allele than those with CC genotype or C allele [12], carriers of the miR-196a2 (rs11614913) T allele were confirmed to associate with susceptibility to HCC among Caucasian populations [15].

Although previous studies analyzed the relationship between different miR-499, miR-196a2 and miR-146a genotypes in different patient populations, their findings were inconsistent, and they did not investigate whether the genotypes affected patients’ clinical characteristics. To determine the role of miRNA SNPs in HCC, we performed a case–control study in which we used a high-resolution melting (HRM) genotyping method to investigate the relationship between the SNPs of four miRNAs (miR-146a C > G, miR-196a2 C > T, miR-301b A > G, and miR-499 C > T) (Additional file 1: Table S1) and HCC. We also analyzed the clinical characteristics of HCC patients with different genotypes to determine the role of miRNA SNPs in HCC.

## Methods

### Study population

The ethics committee of Zhongnan Hospital of Wuhan University has approved the present study (Approval Number 2013059). Informed consent was obtained from all participants at interview, as well as at time of biospecimen collection. We included 314 patients who were diagnosed with HCC at Zhongnan Hospital between 2005 and 2012. All patients had pathologically confirmed HCC and underwent liver resection. The American Joint Committee on Cancer’s TNM (tumor, node, and metastasis) staging system and the Barcelona Clinic Liver Cancer (BCLC) staging system were used to stage patients’ HCC. From these patients we collected 314 formalin-fixed, paraffin-embedded (FFPE) tissue samples (6 mm × 6 mm; about 5 μm thick). The control group consisted of 407 participants randomly selected from healthy individuals enrolled in an HCC screening program who had no history of cancer or chronic disease. Ethylenediaminetetraacetic acid–anticoagulated peripheral blood samples were collected from the control group. Additionally, we collected 39 tumor tissue samples and peripheral blood samples from the same HCC patients. All participants’ hepatitis B surface antigen/hepatitis B virus statuses were assessed by a chemiluminescent enzyme immunoassay. The available preoperative biometrical characteristics and clinical data of the HCC patients and controls are shown in Additional file 1: Table S2.

### DNA extraction

We used commercially available DNA extraction kits to extract genomic DNA from FFPE tissue samples (Paraffin-Embedded Tissue Kit, TaKaRa, Dalian, China) or peripheral blood samples (TIANamp Blood DNA Kit, Tiangen, Beijing, China) according to the manufacturer’s instructions. We used a DU 530 spectrophotometer (Beckman Coulter, Fullerton, CA, USA) to quantify the concentration of DNA; absorbance readings of the DNA extracts at 260 nm indicated that the DNA concentration was about 720.94 μg/mL. The extracted DNA samples were frozen at −20°C without repeated freeze-thawing cycles until subjected to assay.

### SNP genotyping

To genotype the four SNPs, we performed HRM of small amplicons using the LightScanner 32 system (Idaho Technology, Salt Lake City, UT, USA) in tumor and blood samples from HCC patients. We initially tested the concordance between genotypes from 39 paired tumor and blood samples using the k statistic. Then, we investigated the four miRNAS’ SNPs in the population of 314 HCC patients with FFPE samples, and 407 controls with peripheral blood samples. The primers used for the HRM analysis are shown in Additional file 1: Table S3. The amplifications were performed in 10-μL volumes containing 10–20 ng of genomic DNA, 0.16 μM primer, 250 μM of each deoxynucleotide triphosphate, 1.25 μM Mg^2+^, 2 μL of 5× polymerase chain reaction buffer, 1.0 U of polymerase enzyme, and 1× LCGreen Plus + dye (Idaho Technology). Polymerase chain reaction cycling included an initial denaturation at 95°C for 2 min followed by 45 cycles of 15 seconds at 95°C, 15 seconds at the respective annealing temperatures (Additional file 1: Table S3), and 15 seconds at 72°C and final extensions of 30 seconds at 94°C and 30 seconds at 28°C for heteroduplex formation.

For quality control, DNA samples with different known genotypes were included as internal standards in each experiment. A duplicate control without a DNA template was also included in each run to test for contamination and to assess the formation of any primer dimer.

### Statistical analysis

We used the statistical software program SPSS 17.0 for Windows to perform all statistical analyses (SPSS Inc., Chicago, IL). Differences in the clinical characteristics and genotypes between the HCC patients and control participants were evaluated using the Student t-test or one-way ANOVA (for continuous variables) and Pearson chi-square test (for categorical variables). The Pearson chi-square test was also used to determine whether the allele frequencies in the control group were in Hardy-Weinberg equilibrium (HWE). We used logistic regression analysis with adjustment for possible confounders (sex and age) to determine whether the genotypes of the four SNPs were associated with HCC risk; the results are presented as odds ratios (ORs) and 95% confidence intervals (CIs). To compare the clinical characteristics of HCC patients who had different genotypes, we performed a K-independent non-parametric analysis for skewed distribution. We also used SNPStats, a Web-based SNP analysis software program (http://bioinfo.iconcologia.net/snpstats/start.htm), to analyze the four miRNAs’ SNPs. All statistical tests were two-sided, and P values of less than 0.05 or Bonferroni correction–adjusted P values of less than 0.05 were considered statistically significant.

## Results

### Participant characteristics and SNP identification

The HCC patients’ and control participants’ characteristics are shown in Additional file 1: Table S2. We found no significant difference in age (P = 0.252) or sex (P = 0.993) between the HCC patients and controls. Of the HCC patients, 57.5% had stage I disease, 21.1% had stage II disease, 12.7% had stage III disease, and 8.7% had stage IV disease according to the TNM staging system; and 71.1% had stage A disease, 18.1% had stage B disease, 10.4% had stage C disease, and 0.3% had stage D disease according to the BCLC staging system. In the controls, the genotype distributions of the SNPs in miR-196a2 C > T, miR-499 C > T, and miR-301b A > G (rs11614913, rs3746444, and rs384262, respectively) were in HWE, but the SNP in miR-146a C > G (rs2910164) was not (P < 0.001).

**Figure 1:**
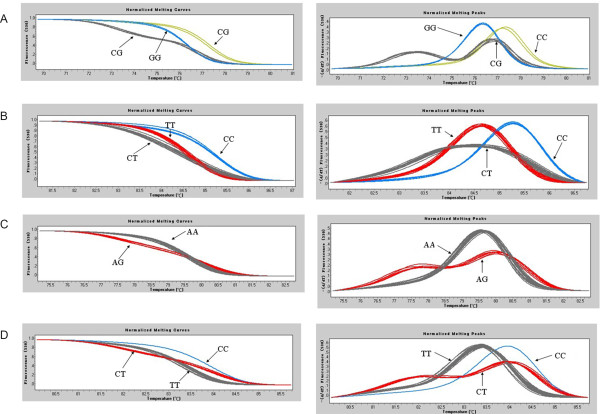
**HRM genotyping of the four SNPs in miRNA.** The normalized melting curves are given in the left column, and the normalized melting peaks are given in the right column. Arrows indicate the genotype. The representative HRM curves of miR-146a C > G (rs2910164), miR-196a2 C > T (rs11614913), miR-301b A > G (rs384262), and miR-499 C > T (rs3746444) are shown in **A**, **B**, **C**, and **D**, respectively.

**Figure 2:**
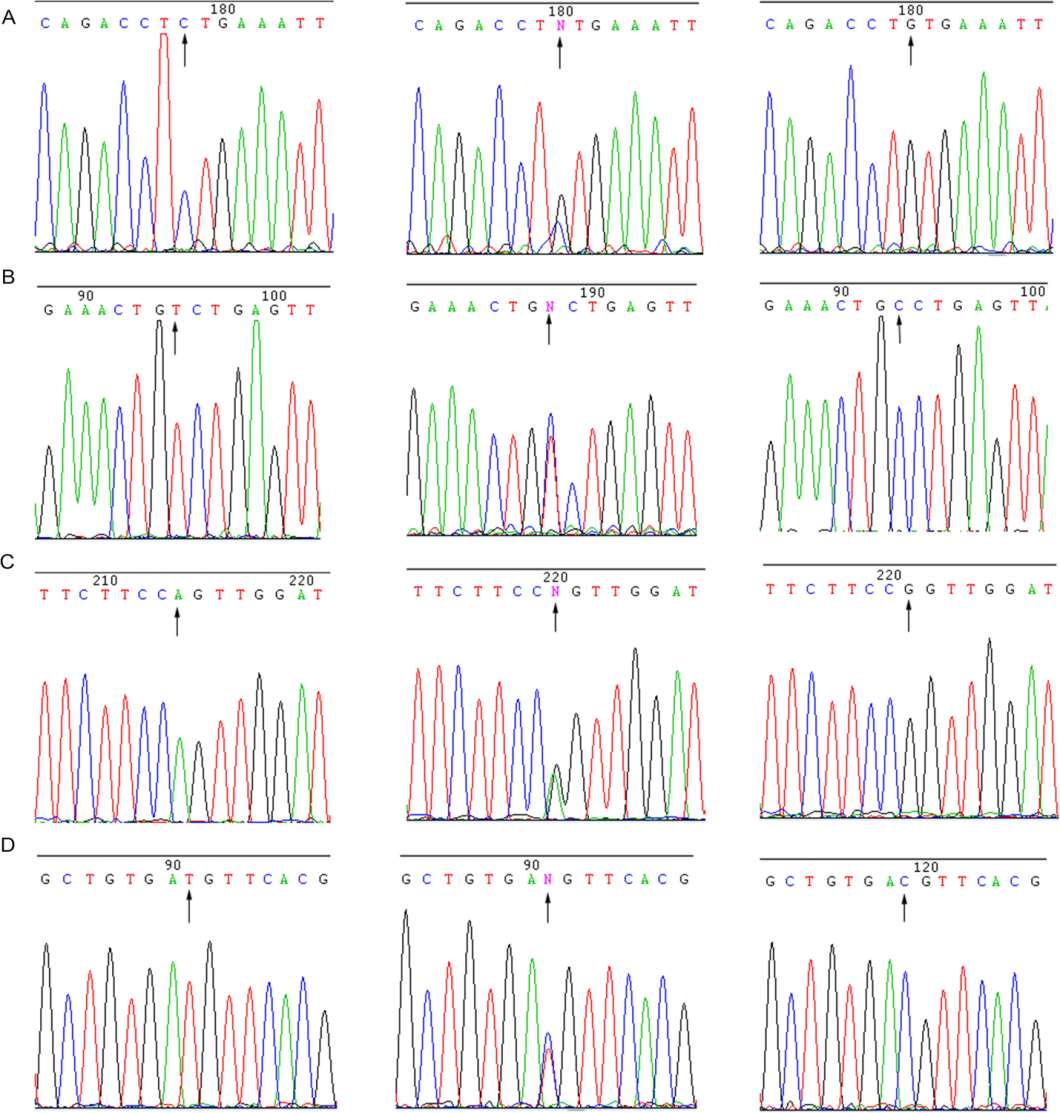
**DNA sequencing of the four SNPs in miRNA.** The three genotypes of miR-146a C > G (rs2910164), miR-196a2 C > T (rs11614913), miR-301b A > G (rs384262), and miR-499 C > T (rs3746444) are shown in **A**, **B**, **C**, and **D**, respectively.

After the melting curves were normalized, different genotypes could be easily distinguished (Figure 1). As expected, the normalized melting peaks revealed that the homozygous samples had clearly defined single peaks for each miRNA SNP (CC or TT peaks for miR-196a2 C > T and miR-499 C > T; AA or GG peaks for miR-301b A > G; CC or GG peaks for miR-146a C > G), and the heterozygous samples had both of the above described peaks for each mircoRNA SNP. The results for 30 DNA samples of each SNP randomly selected for sequencing were fully concordant with HRM, including all mir-499 CC and mir-146a GG genotype samples (Figure 2 and Additional file 1: Table S4).

### Concordance of SNPs in paired tumor and blood samples

It is unclear if genotypes derived from diseased tissue produce the same results as those from paired blood samples. To determine the feasibility of using FFPE tissue samples as a source of genomic DNA in the study, we investigated the concordance between genotypes from 39 paired tumor and blood samples using the κ statistic, which tests the agreement between two paired results. κ > 0.80 indicates a good agreement. Our data demonstrated 100% concordance between the two different specimens, except a discrepancy in one sample for the miR-146a SNP (Table 1, Additional file 1: Table S5).

**Table 1:** **Concordance of SNPs in paired tumor and blood samples**

microRNAs	κ	Asymptotic error	Confidence interval	No. of pairs	No. of nonmatching genotype calls
miR-196a2	1.00	0		39	0
miR-499	1.00	0		39	0
miR-146a	0.96	0.02	(0.95-1.00)	39	1
miR-301b	1.00	0		39	0

### Association of SNPs with HCC risk

After adjustment for confounding factors (sex and age), the results of the risk estimation analysis based on genotype distribution, allele frequency, and genetic model by logistic regression analysis are shown in Table 2. We found no significant difference in the distributions of the SNP in miR-301b A > G (rs384262) between the control participants and HCC patients. However, the distributions of the SNPs in miR-196a2 C > T (rs11614913), miR-499 C > T (rs3746444) and miR146a C > G (rs2910164) in the HCC patients and controls differed significantly (P = 0.017, 7 × 10^−4^ and 0.0015, respectively), which suggests that these SNPs are correlated with HCC risk.

**Table 2:** **Risk estimation based on the distributions of genotype and allele frequency**

microRNA	Model	Genotype	Controls n (%)	HCCs n (%)	AOR ^a^(95% CI)	P	AIC	BIC
miR-196a2	Codominant	TT	121 (29.8)	60 (19.1)	1.00	7e-04	980.3	1003.2
		CT	214 (52.7)	209 (66.6)	1.95 (1.36-2.81)			
		CC	71 (17.5)	45 (14.3)	1.28 (0.79-2.08)			
	Dominant	TT	121 (29.8)	60 (19.1)	1.00	0.0011	982.3	1000.7
		CT + CC	285 (70.2)	254 (80.9)	1.79 (1.25-2.54)			
	Recessive	TT + CT	335 (82.5)	269 (85.7)	1.00	0.26	991.8	1010.1
		CC	71 (17.5)	45 (14.3)	0.79 (0.53-1.19)			
	Overdominant	TT + CC	192 (47.3)	105 (33.4)	1.00	2e-04	979.3	997.6
		CT	214 (52.7)	209 (66.6)	1.77 (1.31-2.41)			
	Addictive				1.20 (0.95-1.52)	0.12	990.6	1008.9
	Allele	T	457 (56.0)	329 (52.0)	1.00	0.156		
		C	357 (44.0)	299 (48.0)	1.16 (0.94-1.43)			
miR-499	Codominant	TT	301 (74.1)	195 (62.1)	1.00	0.0015	982.1	1005
		CT	101 (24.9)	117 (37.3)	1.79 (1.30-2.47)			
		CC	4 (1)	2 (0.6)	0.73 (0.13-4.06)			
	Dominant	TT	301 (74.1)	195 (62.1)	1.00	6e-04	981.2	999.5
		CT + CC	105 (25.9)	119 (37.9)	1.75 (1.27-2.40)			
	Recessive	TT + CT	402 (99.0)	312 (99.4)	1.00	0.57	992.7	1011
		CC	4 (1.0)	2 (0.6)	0.61 (0.11-3.38)			
	Overdominant	TT + CC	305 (75.1)	197 (62.7)	1.00	3e-04	980.2	998.5
		CT	101 (24.9)	117 (37.3)	1.80 (1.30-2.48)			
	Addictive				1.64 (1.21-2.23)	0.0014	982.9	1001.2
	Allele	T	704 (86.0)	507 (81.0)	1.00	0.003		
		C	110 (14.0)	121 (19.0)	1.53 (1.15-2.03)			
miR-146a	Codominant	CC	159 (39.2)	149 (47.5)	1.00	0.017	986.8	1009.7
		CG	244 (60.1)	165 (52.5)	0.71 (0.53-0.96)			
		GG	3 (0.7)	0 (0)	0.00 (0.00-NA)			
	Dominant	CC	159 (39.2)	149 (47.5)	1.00	0.02	987.6	1005.9
		CG + GG	247 (60.8)	165 (52.5)	0.70 (0.52-0.95)			
	Recessive	CC + CG	403 (99.3)	314 (100)	1.00	0.074	989.8	1008.2
		GG	3 (0.7)	0 (0.0)	0.00 (0.00-NA)			
	Overdominant	CC + GG	162 (39.9)	149 (47.5)	1.00	0.033	988.5	1006.8
		CG	244 (60.1)	165 (52.5)	0.72 (0.54-0.97)			
	Addictive				0.69 (0.51-0.93)	0.013	986.9	1005.2
	Allele	C	564 (69.0)	463 (74.0)	1.00	0.065		
		G	250 (31.0)	165 (26.0)	0.80 (0.64-1.01)			
miR-301b	Codominant	AA	319 (78.6)	248 (79.0)	1.00	0.96	994.9	1017.8
		AG	85 (20.9)	65 (20.7)	1.00 (0.69-1.43)			
		GG	2 (0.5)	1 (0.3)	0.70 (0.06-7.78)			
	Dominant	AA	319 (78.6)	248 (79.0)	1.00	0.95	993	1011.3
		AG + GG	87 (21.4)	66 (21.0)	0.99 (0.69-1.42)			
	Recessive	AA + AG	404 (99.5)	313 (99.7)	1.00	0.77	992.9	1011.3
		GG	2 (0.5)	1 (0.3)	0.70 (0.06-7.78)			
	Overdominant	AA + GG	321 (79.1)	249 (79.3)	1.00	0.99	993	1011.3
		AG	85 (20.9)	65 (20.7)	1.00 (0.69-1.44)			
	Addictive				0.98 (0.69-1.40)	0.92	993	1011.3
	Allele	A	725 (89.0)	561 (89.0)	1.00	0.872		
		G	89 (11.0)	67 (11.0)	0.97 (0.70-1.36)			

HCC, hepatocellular carcinoma; AOR, adjusted odds ratio; CI, confidence interval; AIC, Akaike Information Criterion; BIC, Bayesian Information Criterion.

NA, not available.

^a^Adjusted for age and sex.

For the miR-196a2 C > T (rs11614913) polymorphism, the HCC risk of individuals with TT genotype was significantly lower than that of individuals with CT genotype in codominant model (adjusted OR [AOR], 1.95; 95% CI, 1.36-2.81; P = 7 × 10^−4^) and that of individuals with either CT or CC genotype in dominant model (AOR, 1.79; 95% CI, 1.25-2.54; P = 0.0011). We also found that the HCC risk of individuals with CT genotype was significantly higher than that of individuals with either CC or TT genotype in overdominant model (AOR, 1.77; 95% CI, 1.31-2.41; P = 2 × 10^−4^).

For the miR-499 C > T (rs3746444) polymorphism, the HCC risk of individuals with TT genotype was significantly lower than that of individuals with CT genotype in codominant model (AOR, 1.79; 95% CI, 1.30-2.47; P = 0.0015) and that of individuals with either CT or CC genotype (AOR, 1.75; 95% CI, 1.27-2.40; P = 6 × 10^−4^) in dominant model. We also found that the HCC risk of individuals with either CC or TT genotype was significantly lower than that of individuals with CT genotype in overdominant model (AOR, 1.80; 95% CI, 1.30-2.48; P = 3 × 10^−4^). Additionally, the minor C allele of miR-499 (rs3746444) was associated with a higher risk of HCC (AOR, 1.53; 95% CI, 1.15-2.03, P = 0.003).

For the miR146a C > G (rs2910164) polymorphism, the HCC risk of individuals with CG genotype was significantly lower than that of individuals with CC genotype in codominant model (AOR, 0.71; 95% CI, 0.53-0.96; P = 0.017) and that of individuals with either CG or GG genotype in dominant model (AOR, 0.70; 95% CI, 0.52-0.95; P = 0.02). Moreover, the HCC risk of individuals with CG genotype was significantly lower than that of individuals with either CC or GG genotype in overdominant model (AOR, 0.72; 95% CI, 0.54-0.97; P = 0.033).

In addition, the results of a logistic regression analysis were consistent with those of the SNPStats analysis (special analysis of the SNP online software).

### Combined effect of the SNPs associated with HCC risk

To assess the combined effect of the SNPs associated with HCC risk, we performed a combined analysis of the SNPs in miR-196a2 and miR-499. The HCC risk of patients who had both unfavorable genotypes was 3.11 times higher than that of patients who had neither unfavorable genotype (95% CI, 1.89-5.09; P = 7.18 × 10^−6^) (Table 3).

**Table 3:** **Joint effect of unfavorable SNP genotypes associated with hepatocellular carcinoma risk**

No. of unfavorable SNPs ^a^	Controls n (%)	HCCs n (%)	AOR ^b^(95% CI)	P
0	82 (20.2)	39(12.4)	1.00	
1	261 (64.1)	177 (56.4)	1.40 (0.91-2.14)	0.126
2	64 (15.7)	98 (31.2)	3.11 (1.89-5.09)	7.18x10^−6^

AOR, adjusted odds ratio; CI, confidence interval.

^a^Unfavorable genotypes were potentially risk genotypes (CT + CC for miR-196a2 and miR-499).

^b^ORs were adjusted for age and sex.

### SNPs’ effects on HCC patients’ clinical characteristics

We also compared the clinical characteristics of HCC patients who had different microRNA SNP genotypes. The patients with TT, CT, or CC genotype of the miR-196a2 C > T (rs11614913) had significantly different clinical characteristics (Table 4). We found that the activated partial thromboplastin time (APTT) differed among HCC patients with different miR-196a2 C > T genotypes (P = 0.032) by one-way ANOVA analysis, and LSD multiple comparisons indicated that patients with CC genotype had longer APTT than that of patients with CT genotype (37.1 ± 8.0 vs. 33.9 ± 7.3, P = 0.011). For the miR-499 SNP, several patients had CC genotype; therefore, we combined patients with CT or CC genotype into one group. We found that the differences in liver function parameters between patients with TT genotype and patients with either CT or CC genotype differed significantly. Compared with patients who had either CT or CC genotype, patients with TT genotype had slightly lower concentrations of direct bilirubin (P = 0.031), globulin (P = 0.034), γ-glutamyltranspeptidase (P = 0.022), alkaline phosphatase (P = 0.002), and higher cholinesterase (P = 0.028) (Table 5). For the miR-146a SNP, compared to the patients with either CG or GG genotype, patients with CC genotype had higher albumin-to-globulin ratios (P = 0.011) (Additional file 1: Table S6). As regard the miR-301b SNP, neither genotype distributions nor the 34 clinical parameters differed significantly between the HCC patients and control participants.

**Table 4:** **Comparative analysis of the clinical characteristics of hepatocellular carcinoma patients with different miR-196a2 genotypes**

Characteristic	Reference intervals	TT (n = 60)	CT (n = 209)	CC (n = 45)	P
Alanine amiotransferase (U/L)^a^	0-46	36.0 (22.8, 60.8)	39.0 (28.0, 78.3)	39.0 (31.0, 78.5)	0.364
Aspartate aminotransferase, (U/L)^a^	0-46	43.0 (30.0, 62.8)	46.0 (30.3, 85.5)	46.0 (35.0, 72.0)	0.468
Total bilirubin (μmol/L)^a^	0-25	20.7 (16.0, 29.6)	18.2 (13.0, 24.9)	18.3 (14.9, 24.7)	0.186
Direct bilirubin (μmol/L)^a^	0-7	5.9 (4.0, 8.5)	4.8 (3.5, 6.8)	5.3 (3.7, 7.1)	0.134
Indirect bilirubin (μmol/L)^a^	1.5-18	15.2 (11.7, 20.0)	13.7(9.6,18.9)	12.7 (9.5, 17.1)	0.227
Total protein (g/L)^a^	60-80	69.5 (64.4, 73.8)	68.1 (62.2, 74.0)	67.1 (61.7, 71.3)	0.215
Albumin (g/L)^b^	35-55	40.3 ± 5.0	40.4 ± 6.0	38.8 ± 6.8	0.266
Globulin (g/L)^a^	20-30	29.5 (24.8, 31.9)	27.5 (23.5, 30.6)	26.7 (23.2, 29.8)	0.112
Albumin/Globulin^a^	1.5-2.5	1.4 (1.3, 1.6)	1.5 (1.3, 1.7)	1.5 (1.2, 1.7)	0.316
γ-glutamyltransferase (U/L)^a^	5-55	60.5 (39.8, 134.5)	62.0 (39.3, 118.0)	67.5 (31.3, 97.5)	0.716
Alkaline phosphatase (U/L)^a^	35-134	93.0 (71.8, 113.3)	102.0 (78.0, 137.0)	91.0 (77.0, 110.8)	0.170
5-Nucleotidase (U/L)^a^	0-10	3.5 (2.0, 5.8)	3.0 (2.0, 6.0)	3.0 (2.0, 4.5)	0.403
Total biliary acid (μmol/L)^a^	0-15	7.4 (4.0, 22.4)	7.1 (3.6, 15.6)	7.1(4.0,14.7)	0.553
Cholinesterase (U/L)^a^	3000-10500	5940.8 ± 2522.3	6074.5 ± 2231.3	6007.5 ± 2498.1	0.927
Pre-albumin (mg/L)^a^	100-400	119.5 (68.8, 157.9)	118.0 (75.0, 175.3)	110.0 (56.0, 172.5)	0.493
Glucose (mmol/L)^a^	3.9-6.2	5.0 (4.4, 6.0)	5.1 (4.6, 5.8)	5.1 (4.4, 5.6)	0.653
Blood urea nitrogen (mmol/L)^a^	1.7-7.2	4.9 (3.5, 5.7)	5.0 (3.8, 6.0)	5.2 (3.3, 6.2)	0.633
Creatinine (μmol/L)^a^	45-117	76.2 (69.1, 83.7)	73.5 (64.1, 84.0)	72.2 (62.7, 83.7)	0.488
Uric acid (μmol/L)^b^	119-417	243.0 ± 77.3	257.3 ± 92.8	251.7 ± 72.9	0.547
Retinol-binding protein (mg/L)^a^	15-70	25.4 (16.1, 34.5)	28.1 (17.4, 35.0)	27.2 (16.3, 35.7)	0.990
Cystatin C (mg/L)^a^	0-1.2	1.1 (0.8, 1.2)	1.0 (0.9, 1.2)	1.0 (0.9, 1.2)	0.999
Carcinoembryonic antigen, (ng/mL)^a^	0-5	2.0 (1.6, 2.9)	2.3 (1.4, 3.5)	2.2 (1.8, 3.3)	0.683
Alpha-fetoprotein (ng/mL)^a^	0-20	124.1 (9.8, 865.8)	130.7 (8.5, 957.9)	382.4 (36.2, 1052.0)	0.116
Ferritin (ng/mL)^a^	0-322	353.0 (138.9, 431.3)	204.5 (116.7, 342.0)	232.9 (174.6, 392.0)	0.281
Cancer antigen 125 (KU/L)^a^	0-35	12.8 (8.7, 38.3)	15.5 (9.6, 32.5)	17.3 (9.9, 58.2)	0.701
Cancer antigen 153 (KU/L)^a^	0-35	11.5 (9.6, 16.0)	9.3 (7.5, 13.6)	10.6 (7.8, 17.2)	0.206
Cancer antigen 199 (KU/L)^a^	0-35	13.3 (8.0, 23.1)	13.3 (6.4, 26.3)	12.8 (6.6, 17.2)	0.779
Prothrombin time (sec)^a^	10.5-13.5	12.0 (11.4, 13.2)	11.9 (11.0, 12.9)	12.2 (11.3, 13.2)	0.462
PT% (%)^a^	80-130	94.5 (84.2, 100.7)	91.3 (81.6, 109.4)	91.0 (77.2, 101.1)	0.603
International standard ratio^a^	0.85-1.15	1.0 (1.0, 1.2)	1.0 (1.0, 1.1)	1.1 (1.0, 1.1)	0.437
D-fibrinogen (g/L)^a^	2-4	2.5 (2.2, 3.2)	2.8 (2.2, 3.5)	2.5 (2.1, 3.1)	0.348
activated partial thrombo -plastin time (sec)^b^	28-40	35.2 ± 7.6	33.9 ± 7.3	37.1 ± 8.0	0.032
Thrombin time (sec)^a^	11-14	14.4 (13.6, 15.7)	14.4 (13.7, 15.4)	14.5(13.7,16.0)	0.872

^a^Data were expressed as median (25th percentile, 75th percentile).

^b^Data were expressed as mean ± SD.

**Table 5:** **Comparative analysis of the clinical characteristics of hepatocellular carcinoma patients with different miR-499 genotypes**

Characteristic	Reference intervals	TT (n = 195)	CT + CC (n = 119)	P
Alanine amiotransferase (U/L)^a^	0-46	38.0 (28.0, 66.0)	39.5 (28.3, 90.3)	0.470
Aspartate aminotransferase, (U/L)^a^	0-46	44.0 (30.0, 68.0)	47.0 (32.3, 90.3)	0.385
Total bilirubin (μmol/L)^a^	0-25	18.7 (13.3, 25.4)	19.1 (14.3, 27.9)	0.615
Direct bilirubin (μmol/L)^a^	0-7	4.7 (3.4, 6.6)	5.5 (4.1, 8.5)	0.031
Indirect bilirubin (μmol/L)^a^	1.5-18	13.9 (9.7,1 8.6)	14.2 (10.0, 19.1)	0.965
Total protein (g/L)^a^	60-80	68.0 (61.5, 73.2)	68.6 (63.6, 74.1)	0.307
Albumin (g/L)^b^	35-55	40.4 ± 6.2	39.8 ± 5.5	0.378
Globulin (g/L)^a^	20-30	27.1 (23.5, 30.3)	29.1 (24.9, 32.1)	0.034
Albumin/Globulin^a^	1.5-2.5	1.5 (1.3, 1.7)	1.4 (1.3, 1.7)	0.099
γ-glutamyltransferase (U/L)^a^	5-55	55.0 (40.0, 99.0)	73.0 (37.0, 191.0)	0.022
Alkaline phosphatase (U/L)^a^	35-134	92.0 (74.0, 120.0)	108.5 (84.5, 136.8)	0.002
5-Nucleotidase (U/L)^a^	0-10	3.0 (2.0, 5.0)	3.0 (2.0, 7.5)	0.236
Total biliary acid (μmol/L)^a^	0-15	6.9 (3.5, 13.9)	9.3 (4.5, 19.4)	0.149
Cholinesterase (U/L)^a^	3000-10500	6264.2 ± 2351.8	5645.5 ± 2225.7	0.028
Pre-albumin (mg/L)^a^	100-400	125.5 (69.7, 168.9)	100.0 (70.0, 176.3)	0.374
Glucose (mmol/L)^a^	3.9-6.2	5.0 (4.5, 5.8)	5.2 (4.7, 5.9)	0.085
Blood urea nitrogen (mmol/L)^a^	1.7-7.2	5.0 (3.5, 5.8)	5.1 (3.9, 6.4)	0.187
Creatinine (μmol/L)^a^	45-117	73.8 (65.5, 84.0)	73.8 (61.9, 83.7)	0.187
Uric acid (μmol/L)^b^	119-417	252.0 ± 87.4	256.9 ± 87.3	0.642
Retinol-binding protein (mg/L)^a^	15-70	28.0 (17.9, 34.6)	27.9 (15.9, 38.2)	0.695
Cystatin C (mg/L)^a^	0-1.2	1.1 (0.9, 1.2)	1.0 (0.9, 1.3)	0.782
Carcinoembryonic antigen,(ng/mL)^a^	0-5	2.1 (1.4, 3.4)	2.3 (1.6, 1.9)	0.365
Alpha-fetoprotein (ng/mL)^a^	0-20	181.7 (10.9, 1000.0)	99.2 (8.4, 691.2)	0.164
Ferritin (ng/mL)^a^	0-322	196.8 (126.7, 361.2)	277.9 (161.0, 424.6)	0.212
Cancer antigen 125 (KU/L)^a^	0-35	15.2 (10.1, 31.7)	16.0 (8.6, 45.5)	0.707
Cancer antigen 153 (KU/L)^a^	0-35	10.4 (7.6, 14.1)	9.6 (7.3, 14.3)	0.720
Cancer antigen 199 (KU/L)^a^	0-35	13.3 (7.1, 23.7)	12.4 (6.6, 26.8)	0.585
Prothrombin time (sec)^a^	10.5-13.5	12.0 (11.3, 12.8)	11.9 (10.9, 13.2)	0.875
PT% (%)^a^	80-130	91.3 (82.0, 104.0)	93.5 (79.8, 109.0)	0.744
International standard ratio^a^	0.85-1.15	1.0 (1.0, 1.1)	1.0 (1.0, 1.2)	0.815
D-fibrinogen (g/L)^a^	2-4	2.7 (2.2, 3.4)	2.6 (2.3, 3.3)	0.724
activated partial thrombo-plastin time (sec)^b^	28-40	34.8 ± 7.3	34.3 ± 7.9	0.568
Thrombin time (sec)^a^	11-14	14.4 (13.8, 15.5)	14.5 (13.5, 15.5)	0.886

^a^Data were expressed as median (25th percentile, 75th percentile).

^b^Data were expressed as mean ± SD.

### Stratified analysis

To examine whether the genotype distributions of the four SNPs are correlated with patients’ hepatitis B surface antigen/hepatitis B virus status, we divided the HCC patients into two groups: hepatitis B virus–positive (n = 243) and hepatitis B virus–negative (n = 49). We found no significant difference in the genotype distributions of the four SNPs between hepatitis B virus–positive and hepatitis B virus–negative HCC patients (P > 0.05). We also found no significant association between the TNM or BCLC tumor stage and the HCC risk of patients with different genotypes (P > 0.05).

## Discussion and conclusions

Because the findings of previous studies regarding the roles of miRNA SNPs in HCC were inconclusive or inconsistent, they seem to be one of the underpinnings of the rationale for guiding us in the present study. We used HRM methods to detect the SNPs of miR-196a2 C > T, miR-499C > T, miR-146a C > G, and miR-301b A > G in HCC. HRM has been developed for the detection of DNA sequence variants and it was applied first for genotyping in 2003 [34], which is a closed-tube method in which the PCR amplification and can be analyzed in the same well to detect mutations [35, 36]. HRM does not require post-PCR separation, significant cost savings are achieved and becomes the most important mutation detection technique and has been widely applied in the polymorphisms detection and epigenetics studies [22, 37, 38]. HRM analysis was an efficient tool for studies of SNPs in miRNAs’ SNPs analysis in acute leukemia [39] and colorectal cancers [22] just two years ago. For evaluating the sensitivity and specificity of SNP scanning by HRM, Reed and Wittwer confirmed that the PCR products of 300 bp or less, all the heterozygous and wild-type cases were correctly called without error. Between 400 and 1000 bp with the mutation centered, the sensitivity and specificity were 96.1% and 99.4% [40], which indicated that HRM method would be made our findings more robust than the previous studies in HCC. We used both logistic regression analysis and SNPStats to assess the association between the four SNPs and HCC risk, we found that the SNPs in miR-196a2 C > T, miR-499 C > T and miR-146a C > G, but not in miR-301b A > G, in HCC patients and control participants differed significantly. Given that the C alleles of miR-196a2 and miR-499 are relatively scarce in Asian populations [11–13, 15, 16, 18, 19, 33], we combined the CT and CC as a dominant genotype model and found that the HCC risk of participants with the CC or CT genotype was significantly higher than that of participants with the TT genotype.

Our study demonstrates that miR-196a2 C > T and miR-499 C > T increase HCC risk. The HCC risks of participants who had the variant heterozygous CT genotype of miR-196a2 or miR-499 were significantly higher than those of participants who had the wild-type homozygous TT genotype of miR-196a2 (AOR, 1.95; 95% CI, 1.36–2.81) or miR-499 (AOR, 1.79; 95% CI, 1.30–2.47). These results are in agreement with those reported for Chinese HCC patients. In male Chinese patients with HBV infection, the risk of HCC was significantly higher in patients with the CC genotype or carrying C allele for miR-196a2 than those with the TT genotype or T allele [12]. Similarly, carriers of miRNA-499 CC were associated with a higher risk of HCC in Chinese population [13]. Our result also supports a previous report that common genetic polymorphisms in miR-196a2 and miR-499 may contribute to breast cancer susceptibility (OR, 1.23; 95% CI, 1.02-1.48 for miR-196a2; and OR, 1.25; 95% CI, 1.02-1.51 for miR-499 in a dominant genetic model) [18]. Additionally, another study found that the miR-499C > T in a dominant genetic model increased the cervical squamous cell carcinoma risk (OR, 1.78; 95% CI, 1.24–2.56) [23].

Our results also suggested that the HCC risks of participants with CG genotype of miR-146a were lower than those with TT genotype (AOR, 0.71; 95% CI, 0.53-0.96). A recent meta-analysis indicated a similar result that the miR-146a C > G variant was associated with a decreased HCC risk among Asian populations [15]. However, several studies reported that miR-146a C > G was not associated with the risk of HCC [13, 33]. Besides, miR-146a could promote cell proliferation and colony formation in NIH/3T3 [14]. In one study, men with the GG genotype were twice as susceptible to HCC as those with the CC genotype (OR, 2.016; 95% CI, 1.056-3.848; P = 0.034); the researchers also found that the mature miR-146a production of the G-allelic miR-146a precursor was higher than that of the C-allelic miR-146a precursor [14]. In addition to HCC, miR-146a C > G has been associated with cervical squamous cell carcinoma [23], familial breast/ovarian cancer [24] and thyroid carcinoma [25]. It should be noted that the genotype distribution of miR-146a C > G (rs2910164) was not in HWE. In line with our data, Chu et al. found that miRNA 149 (rs2292832) deviated from HWE in healthy control participants [27], and another study of 107,000 genotypes generated from 443 SNPs revealed that the genotype distributions of 36 of 313 assays (11.5%) were not in HWE, and the reason for the remaining 10 SNPs deviated from HWE was unclear [41]. The limitation of this study is that the reason for the nonconformity of miR-146a C > G (rs2910164) genotypes to HWE in healthy control participants has not been clarified, further investigation of miR-146a function in HCC needs to be carried in the future.

In the present study, the distributions of the miR-301b genotypes in HCC patients and control participants did not differ significantly. The SNPs of miR-196a2 C > T, miR-499 C > T, and miR146a C > G are all located in 3p mature miRNA regions and may influence both the binding of target mRNAs to 3p and the pre-miRNA maturation of 5p and 3p. However, the SNP of miR-301b A > G is located in the miRNA flanking region. This may explain the lack of a significant difference in the distributions of the miR-301b genotypes between the two groups; perhaps this SNP did not change the maturation of the miR-301b and thus did not influence the binding of target mRNAs to 3p.

In addition, the clinical characteristics of patients with different miRNA genotypes were different, and these characteristics were correlated with different genotypes. The patients with CC genotype of the miR-196a2 C > T (rs11614913) had significantly longer APTT. For the miR-499 C > T (rs3746444), we also demonstrated that the patients with TT genotype had lower direct bilirubin, globulin, γ-glutamyltranspeptidase, alkaline phosphatase, and higher cholinesterase. We firstly verified the differences in coagulation function and liver function parameters between patients with TT genotype of the miR-196a2 C > T (rs11614913) or miR-499 C > T (rs3746444) and the patients with either CT or CC genotype differed significantly. The increased total bilirubin (P < 0.0001) and decreased albumin (P < 0.0001) were related to poor prognosis in patients with HCC [42]. On the other hand, preoperative alkaline phosphatase level could be utilized to monitor and predict recurrence in high risk HCC patients [43] and preoperative cholinesterase levels contributed important information in predicting postoperative outcome after hepatic resection for HCC, and cholinesterase ≤ 5,900 U/L independently predicted the risk of morbidity [44]. These results implied that miR-196a2 C > T (rs11614913) and miR-499 C > T (rs3746444) were possibly related to the prognosis and outcome in patients with HCC.

Our findings suggest that miR-196a2C > T and miR-499C > T increased HCC risk, and different genotypes of the SNPs in three miRNAs affected the clinical laboratory characteristics of HCC patients. It is the first study to demonstrate the relationship between different genotypes and the clinical laboratory characteristics of HCC patients. Future studies should identify the specific mechanism underlying miR-196a2C > T and miR-499 C > T genotypes as well as altered clinical laboratory characteristics, which should provide valuable information facilitating the early detection and diagnosis of HCC.

It is well known that cancer tissues show frequent mutations even at SNP sites and the sequence variations in tumor tissues maybe be different from those of normal blood samples, which will almost certainly lead to questions of how to justify the tissue-with-blood comparisons. However, in this study, we compared the reliability of genetic studies done on biobanks comprised of FFPE autopsy tissue with banks of blood samples from the same donors, and investigated the association of four miRNAs’ SNPs with HCC risk. Our data suggested that the genotypes of miRNA’s SNPs were almost identical in HCC tissue and peripheral blood samples from the same patients (n = 39). The similar results were performed by Sjöholm et al., which showed that DNA from all plasma (n = 30, HCC patients) and serum (n = 1, additional patient) samples gave identical genotyping results as obtained from tissue DNA from the same subject by comparison of archival plasma and FFPE tissue for genotyping in HCC, who also reported 100% each-way matching [45].

Finally, our results were based on a small sample size. Further validation of these findings is warranted in larger studies. We will collect more FFPE tissue and blood samples from HCC patients to further address the clinical utility of the miRNA SNPs for the risk prediction of HCC.

## Electronic supplementary material

Additional file 1: Table S1: Characteristics of the four single-nucleotide polymorphisms (SNPs); **Table S2.** Characteristics of hepatocellular carcinoma (HCC) patients and controls; **Table S3.** Primers used for high-resolution melting (HRM) analysis; **Table S4.** Primers used for DNA sequencing; **Table S5.** Comparison of the four miRNAs’ SNPs in paired tumor and blood samples; **Table S6.** Comparative analysis of the clinical characteristics of hepatocellular carcinoma patients with different miR-146a genotypes. (DOC 218 KB)

## Abbreviations

HCC: Hepatocellular carcinoma
SNP: Single-nucleotide polymorphism
miRNA: MicroRNA
HRM: High-resolution melting
FFPE: Formalin-fixed, paraffin-embedded
HWE: Hardy-weinberg equilibrium
TNM: Tumor, node, metastasis
BCLC: Barcelona clinic liver cancer.

## References

[1] Fares N, Peron JM (2013). Epidemiology, natural history, and risk factors of hepatocellular carcinoma. Rev Prat.

[2] Schwartz M, Roayaie S, Konstadoulakis M (2007). Strategies for the management of hepatocellular carcinoma. Nat Clin Pract Oncol.

[3] Qi J, Wang J, Katayama H, Sen S, Liu SM (2013). Circulating microRNAs (cmiRNAs) as novel potential biomarkers for hepatocellular carcinoma. Neoplasma.

[4] El-Serag HB (2012). Epidemiology of viral hepatitis and hepatocellular carcinoma. Gastroenterology.

[5] Tanaka M, Katayama F, Kato H, Tanaka H, Wang J, Qiao YL, Inoue M (2011). Hepatitis B and C virus infection and hepatocellular carcinoma in China: a review of epidemiology and control measures. J Epidemiol.

[6] Bartel DP (2004). MicroRNAs: genomics, biogenesis, mechanism, and function. Cell.

[7] Wang J, Sen S (2011). MicroRNA functional network in pancreatic cancer: from biology to biomarkers of disease. J Biosci.

[8] Ambros V (2003). MicroRNA pathways in flies and worms: growth, death, fat, stress, and timing. Cell.

[9] Esquela-Kerscher A, Slack FJ (2006). Oncomirs - microRNAs with a role in cancer. Nat Rev Cancer.

[10] Horikawa Y, Wood CG, Yang H, Zhao H, Ye Y, Gu J, Lin J, Habuchi T, Wu X (2008). Single nucleotide polymorphisms of microRNA machinery genes modify the risk of renal cell carcinoma. Clin Cancer Res.

[11] Hu Z, Chen J, Tian T, Zhou X, Gu H, Xu L, Zeng Y, Miao R, Jin G, Ma H, Chen Y, Shen H (2008). Genetic variants of miRNA sequences and non-small cell lung cancer survival. J Clin Invest.

[12] Qi P, Dou TH, Geng L, Zhou FG, Gu X, Wang H, Gao CF (2010). Association of a variant in MIR 196A2 with susceptibility to hepatocellular carcinoma in male Chinese patients with chronic hepatitis B virus infection. Hum Immunol.

[13] Xiang Y, Fan S, Cao J, Huang S, Zhang LP (2012). Association of the microRNA-499 variants with susceptibility to hepatocellular carcinoma in a Chinese population. Mol Biol Rep.

[14] Xu T, Zhu Y, Wei QK, Yuan Y, Zhou F, Ge YY, Yang JR, Su H, Zhuang SM (2008). A functional polymorphism in the miR-146a gene is associated with the risk for hepatocellular carcinoma. Carcinogenesis.

[15] Xu Y, Li L, Xiang X, Wang H, Cai W, Xie J, Han Y, Bao S, Xie Q (2013). Three common functional polymorphisms in microRNA encoding genes in the susceptibility to hepatocellular carcinoma: A systematic review and meta-analysis. Gene.

[16] Guo J, Jin M, Zhang M, Chen K (2012). A genetic variant in miR-196a2 increased digestive system cancer risks: a meta-analysis of 15 case–control studies. PLoS One.

[17] Gao LB, Bai P, Pan XM, Jia J, Li LJ, Liang WB, Tang M, Zhang LS, Wei YG, Zhang L (2011). The association between two polymorphisms in pre-miRNAs and breast cancer risk: a meta-analysis. Breast Cancer Res Treat.

[18] Hu Z, Liang J, Wang Z, Tian T, Zhou X, Chen J, Miao R, Wang Y, Wang X, Shen H (2009). Common genetic variants in pre-microRNAs were associated with increased risk of breast cancer in Chinese women. Hum Mutat.

[19] Peng S, Kuang Z, Sheng C, Zhang Y, Xu H, Cheng Q (2010). Association of microRNA-196a-2 gene polymorphism with gastric cancer risk in a Chinese population. Dig Dis Sci.

[20] Kogo R, Mimori K, Tanaka F, Komune S, Mori M (2011). Clinical significance of miR-146a in gastric cancer cases. Clin Cancer Res.

[21] Zhu L, Chu H, Gu D, Ma L, Shi D, Zhong D, Tong N, Zhang Z, Wang M (2012). A functional polymorphism in miRNA-196a2 is associated with colorectal cancer risk in a Chinese population. DNA Cell Biol.

[22] Vinci S, Gelmini S, Mancini I, Malentacchi F, Pazzagli M, Beltrami C, Pinzani P, Orlando C (2013). Genetic and epigenetic factors in regulation of microRNA in colorectal cancers. Methods.

[23] Zhou B, Wang K, Wang Y, Xi M, Zhang Z, Song Y, Zhang L (2011). Common genetic polymorphisms in pre-microRNAs and risk of cervical squamous cell carcinoma. Mol Carcinog.

[24] Shen J, Ambrosone CB, DiCioccio RA, Odunsi K, Lele SB, Zhao H (2008). A functional polymorphism in the miR-146a gene and age of familial breast/ovarian cancer diagnosis. Carcinogenesis.

[25] Jazdzewski K, Murray EL, Franssila K, Jarzab B, Schoenberg DR, de la Chapelle A (2008). Common SNP in pre-miR-146a decreases mature miR expression and predisposes to papillary thyroid carcinoma. Proc Natl Acad Sci U S A.

[26] Permuth-Wey J, Thompson RC, Burton Nabors L, Olson JJ, Browning JE, Madden MH, Ann Chen Y, Egan KM (2011). A functional polymorphism in the pre-miR-146a gene is associated with risk and prognosis in adult glioma. J Neurooncol.

[27] Chu YH, Tzeng SL, Lin CW, Chien MH, Chen MK, Yang SF (2012). Impacts of microRNA gene polymorphisms on the susceptibility of environmental factors leading to carcinogenesis in oral cancer. PLoS One.

[28] Wei W, Hu Z, Fu H, Tie Y, Zhang H, Wu Y, Zheng X (2012). MicroRNA-1 and microRNA-499 downregulate the expression of the ets1 proto-oncogene in HepG2 cells. Oncol Rep.

[29] Zhu K, Pan Q, Zhang X, Kong LQ, Fan J, Dai Z, Wang L, Yang XR, Hu J, Wan JL, Zhao YM, Tao ZH, Chai ZT, Zeng HY, Tang ZY, Sun HC, Zhou J (2013). MiR-146a enhances angiogenic activity of endothelial cells in hepatocellular carcinoma by promoting PDGFRA expression. Carcinogenesis.

[30] Tomokuni A, Eguchi H, Tomimaru Y, Wada H, Kawamoto K, Kobayashi S, Marubashi S, Tanemura M, Nagano H, Mori M, Doki Y (2011). miR-146a suppresses the sensitivity to interferon-alpha in hepatocellular carcinoma cells. Biochem Biophys Res Commun.

[31] Jiang J, Gusev Y, Aderca I, Mettler TA, Nagorney DM, Brackett DJ, Roberts LR, Schmittgen TD (2008). Association of MicroRNA expression in hepatocellular carcinomas with hepatitis infection, cirrhosis, and patient survival. Clin Cancer Res.

[32] Zhang X, Daucher M, Armistead D, Russell R, Kottilil S (2013). MicroRNA expression profiling in HCV-infected human hepatoma cells identifies potential anti-viral targets induced by interferon-alpha. PLoS One.

[33] Hu M, Zhao L, Hu S, Yang J (2013). The association between two common polymorphisms in MicroRNAs and hepatocellular carcinoma risk in Asian population. PLoS One.

[34] Wittwer CT, Reed GH, Gundry CN, Vandersteen JG, Pryor RJ (2003). High-resolution genotyping by amplicon melting analysis using LCGreen. Clin Chem.

[35] Vossen RH, Aten E, Roos A, den Dunnen JT (2009). High-resolution melting analysis (HRMA): more than just sequence variant screening. Hum Mutat.

[36] Lin CW, Er TK, Tsai FJ, Liu TC, Shin PY, Chang JG (2010). Development of a high-resolution melting method for the screening of Wilson disease-related ATP7B gene mutations. Clin Chim Acta.

[37] Li SW, Lin K, Ma P, Zhang ZL, Zhou YD, Lu SY, Zhou X, Liu SM (2013). FADS gene polymorphisms confer the risk of coronary artery disease in a Chinese Han population through the altered desaturase activities: based on high-resolution melting analysis. PLoS One.

[38] Liu SM, Xu FX, Shen F, Xie Y (2012). Rapid genotyping of APOA5–1131 T > C polymorphism using high resolution melting analysis with unlabeled probes. Gene.

[39] Lin PC, Liu TC, Chang CC, Chen YH, Chang JG (2012). High-resolution melting (HRM) analysis for the detection of single nucleotide polymorphisms in microRNA target sites. Clin Chim Acta.

[40] Reed GH, Wittwer CT (2004). Sensitivity and specificity of single-nucleotide polymorphism scanning by high-resolution melting analysis. Clin Chem.

[41] Hosking L, Lumsden S, Lewis K, Yeo A, McCarthy L, Bansal A, Riley J, Purvis I, Xu CF (2004). Detection of genotyping errors by Hardy-Weinberg equilibrium testing. Eur J Hum Genet.

[42] Kinoshita A, Onoda H, Imai N, Iwaku A, Oishi M, Tanaka K, Fushiya N, Koike K, Nishino H, Matsushima M, Saeki C, Tajiri H (2013). The Glasgow Prognostic Score, an inflammation based prognostic score, predicts survival in patients with hepatocellular carcinoma. BMC Cancer.

[43] Yu MC, Chan KM, Lee CF, Lee YS, Eldeen FZ, Chou HS, Lee WC, Chen MF (2011). Alkaline phosphatase: does it have a role in predicting hepatocellular carcinoma recurrence?. J Gastrointest Surg.

[44] Donadon M, Cimino M, Procopio F, Morenghi E, Montorsi M, Torzilli G (2013). Potential role of cholinesterases to predict short-term outcome after hepatic resection for hepatocellular carcinoma. Updates Surg.

[45] Sjöholm MI, Hoffmann G, Lindgren S, Dillner J, Carlson J (2005). Comparison of archival plasma and formalin-fixed paraffin-embedded tissue for genotyping inhepatocellular carcinoma. Cancer Epidemiol Biomarkers Prev.

[CR46] The pre-publication history for this paper can be accessed here:http://www.biomedcentral.com/1471-2407/14/643/prepub

