# Supplementary material for: High-resolution melting analysis reveals genetic polymorphisms in MicroRNAs confer hepatocellular carcinoma risk in Chinese patients
Source: BMC Cancer. 2014 Aug 31;14:643. doi: 10.1186/1471-2407-14-643 (PMC4161871; doi:10.1186/1471-2407-14-643)
Supplement: Supplementary file 1 — Additional file 1: Table S1: Characteristics of the four single-nucleotide polymorphisms (SNPs); Table S2. Characteristics of hepatocellular carcinoma (HCC) patients and controls; Table S3. Primers used for high-resolution melting (HRM) analysis; Table S4. Primers used for DNA sequencing; Table S5. Comparison of the four miRNAs’ SNPs in paired tumor and blood samples; Table S6. Comparative analysis of the clinical characteristics of hepatocellular carcinoma patients with different miR-146a genotypes. (DOC 218 KB) [file 12885_2014_4818_MOESM1_ESM.doc]

**Additional file 1: Table S1.** Characteristics of the four single-nucleotide polymorphisms (SNPs)

| miRNA | SNP ID | Chromosome | SNP Location | Allele | Location | MAF |
| --- | --- | --- | --- | --- | --- | --- |
| miR-196a2 | rs11614913 | Chr12 | 54385599 | C/T | In-mature | T=0.3878 |
| miR-499 | rs3746444 | Chr20 | 33578215 | C/T | In-seed | C/G=0.1809 |
| miR-146a | rs2910164 | Chr5 | 159912418 | C/G | In-seed | C=0.3814 |
| miR-301b | Rs384262 | Chr22 | 22012772 | A/G | In flanking region | G=0.4245 |

ID, identification; MAF, minor allele frequency.

**Additional file 1: Table S2.** Characteristics of hepatocellular carcinoma (HCC) patients and controls

| **Characteristic** | **HCC Patients** | **Controls** | ***P*** |
| --- | --- | --- | --- |
| Total no. | 314 | 407 |  |
| Mean age ± SD, years | 50.7±12.6 | 49.6±13.5 | 0.252 |
| Male sex | 263 (83.8) | 341 (83.8) | 0.993 |
| Smoking | 122 (38.9) | NA |  |
| Drinking | 86 (27.4) | NA |  |
| HBsAg status |  |  |  |
| HBsAg-positive | 243 (83.2) | 0 | 0.0 |
| HBsAg-negative | 49 (16.8) | 407 (100.0) |  |
| NA | 22 |  |  |
| Tumor size |  |  |  |
| <5 cm | 84 (28.7) |  |  |
| ≥5 cm | 209 (71.3) |  |  |
| NA | 21 |  |  |
| Tumor size |  |  |  |
| <3 cm | 31 (10.6) |  |  |
| ≥3 cm | 262 (89.4) |  |  |
| NA | 21 |  |  |
| TNM stage |  |  |  |
| Ⅰ | 172 (57.5) |  |  |
| Ⅱ | 63 (21.1) |  |  |
| Ⅲ | 38 (12.7) |  |  |
| Ⅳ | 26 (8.7) |  |  |
| NA | 15 |  |  |
| BCLC stage |  |  |  |
| A | 212 (71.1) |  |  |
| B | 54 (18.1) |  |  |
| C | 31 (10.4) |  |  |
| D | 1 (0.3) |  |  |
| NA | 16 |  |  |

Note: All data are no. of patients (%) unless otherwise indicated.

SD, standard deviation; NA, not available; HBsAg, hepatitis B surface antigen;
TNM, tumor, node, metastasis; BCLC, Barcelona Clinic Liver Cancer.

**Additional file 1: Table S3.** Primers used for high-resolution melting (HRM) analysis

| **microRNAs** | **Primers (5’ →3’)** | **Amplicon Length** | **Annealing Temperature** | **HRM Temperature Range** |
| --- | --- | --- | --- | --- |
| miR-196a2 | Forward: TGAACTCGGCAACAAGAAAC Reverse: GGTAGGAGTGGGAGAGGT | 81bp | 59ºC | 65ºC-88ºC |
| miR-499 | Forward: CAGCGTAGGGACGGGAA Reverse: CTTGCAGTGATGTTTAACTCCTC | 72bp | 57ºC | 70ºC-85ºC |
| miR-146a | Forward: GTTGTGTCAGTGTCAGACCT Reverse: TATCCCAGCTGAAGAACTGAATTT | 47bp | 59ºC | 70ºC-83ºC |
| miR-301b | Forward: AGGTGACCACCTCCATT Reverse: ACCTGATCCAAGGAGGG | 50bp | 57ºC | 72ºC-85ºC |

**Additional file 1: Table S4.** Primers used for DNA sequencing

| **microRNA** | **Forward Primers (5’→3’)** | **Reverse Primers (5’→3’)** | **Amplicon Length** |
| --- | --- | --- | --- |
| miR-196a2 | AACCCCCTTCCCTTCTCCTC | GCTTGTCCTCCTTGGTCTGC | 257bp |
| miR-499 | CCTTCGCTGTCTCCCATCA | CGTAAGAAGGCAGCATCG | 328bp |
| miR-146a | TAGACCTGGTACTAGGAAGC | TTTCTCACAGGAACTCACAC | 345bp |
| miR-301b | GCCCTGCCTCTCCTACTCTT | TTCCTGCTCTGGATAAAACC | 289bp |

**Additional file 1:** Table S5. Comparison of the four miRNAs’ SNPs in paired tumor and blood samples

| Patient | Sex | Age | Tumor Stage | | Sample | Genotype | | | | |
| --- | --- | --- | --- | --- | --- | --- | --- | --- | --- | --- |
|  |  |  | TNM | BCLC |  | miR-  196a2 | miR-  499 | miR-  146a | | miR-  301b |
| 1 | Male | 42 | Ⅳa | C | Tissue | CT | TT | CG | AA | |
|  |  |  |  |  | Blood | CT | TT | CG | AA | |
| 2 | Male | 64 | Ⅱ | B | Tissue | TT | TT | CC | AA | |
|  |  |  |  |  | Blood | TT | TT | CC | AA | |
| 3 | Male | 29 | Ⅱ | B | Tissue | TT | TT | CG | AA | |
|  |  |  |  |  | Blood | TT | TT | CG | AA | |
| 4 | Male | 41 | Ⅱ | B | Tissue | CT | CT | CC | AA | |
|  |  |  |  |  | Blood | CT | CT | CC | AA | |
| 5 | Male | 60 | Ⅰ | A | Tissue | CT | TT | CG | AA | |
|  |  |  |  |  | Blood | CT | TT | CG | AA | |
| 6 | Male | 23 | Ⅰ | B | Tissue | CT | CT | CC | AG | |
|  |  |  |  |  | Blood | CT | CT | CC | AG | |
| 7 | Male | 34 | Ⅱ | B | Tissue | TT | CT | CG | AG | |
|  |  |  |  |  | Blood | TT | CT | CG | AG | |
| 8 | Male | 55 | Ⅰ | B | Tissue | CT | TT | CG | AA | |
|  |  |  |  |  | Blood | CT | TT | CG | AA | |
| 9 | Male | 67 | Ⅰ | A | Tissue | CT | CT | CG | AA | |
|  |  |  |  |  | Blood | CT | CT | CG | AA | |
| 10 | Male | 31 | Ⅱ | B | Tissue | CT | TT | CG | AA | |
|  |  |  |  |  | Blood | CT | TT | CG | AA | |
| 11 | Female | 34 | Ⅲb | C | Tissue | CT | TT | CG | AA | |
|  |  |  |  |  | Blood | CT | TT | CG | AA | |
| 12 | Male | 52 | Ⅱ | B | Tissue | CC | TT | CG | AA | |
|  |  |  |  |  | Blood | CC | TT | CG | AA | |
| 13 | Female | 63 | Ⅰ | A | Tissue | CT | CT | CG | AG | |
|  |  |  |  |  | Blood | CT | CT | CG | AG | |
| 14 | Male | 58 | Ⅱ | B | Tissue | CT | CT | CG | AA | |
|  |  |  |  |  | Blood | CT | CT | CG | AA | |
| 15 | Male | 68 | Ⅰ | A | Tissue | CT | CT | CG | AG | |
|  |  |  |  |  | Blood | CT | CT | CG | AG | |
| 16 | Male | 37 | Ⅱ | B | Tissue | CT | CT | CC | AA | |
|  |  |  |  |  | Blood | CT | CT | CC | AA | |
| 17 | Male | 48 | Ⅰ | A | Tissue | CT | CT | CC | AA | |
|  |  |  |  |  | Blood | CT | CT | CC | AA | |
| 18 | Male | 33 | Ⅰ | A | Tissue | TT | CT | CG | AA | |
|  |  |  |  |  | Blood | TT | CT | CG | AA | |
| **19** | **Male** | **50** | **Ⅰ** | **A** | **Tissue** | **TT** | **CT** | **CG** | **AA** | |
|  |  |  |  |  | **Blood** | **TT** | **CT** | **CC** | **AA** | |
| 20 | Male | 53 | Ⅰ | B | Tissue | CT | CT | CC | AG | |
|  |  |  |  |  | Blood | CT | CT | CC | AG | |
| 21 | Male | 60 | Ⅳa | C | Tissue | CT | TT | CG | AA | |
|  |  |  |  |  | Blood | CT | TT | CG | AA | |
| 22 | Male | 43 | Ⅱ | B | Tissue | CT | TT | CC | AG | |
|  |  |  |  |  | Blood | CT | TT | CC | AG | |
| 23 | Male | 50 | Ⅲa | B | Tissue | CT | TT | CG | AA | |
|  |  |  |  |  | Blood | CT | TT | CG | AA | |
| 24 | Female | 59 | Ⅱ | B | Tissue | CT | TT | CC | AA | |
|  |  |  |  |  | Blood | CT | TT | CC | AA | |
| 25 | Male | 55 | Ⅰ | A | Tissue | CT | TT | CC | AA | |
|  |  |  |  |  | Blood | CT | TT | CC | AA | |
| 26 | Male | 24 | Ⅱ | B | Tissue | CT | CT | CC | AA | |
|  |  |  |  |  | Blood | CT | CT | CC | AA | |
| 27 | Female | 48 | Ⅳb | C | Tissue | CT | CT | CG | AA | |
|  |  |  |  |  | Blood | CT | CT | CG | AA | |
| 28 | Male | 49 | ⅢA | B | Tissue | CT | TT | CC | AA | |
|  |  |  |  |  | Blood | CT | TT | CC | AA | |
| 29 | Female | 38 | Ⅰ | A | Tissue | CT | TT | CC | AA | |
|  |  |  |  |  | Blood | CT | TT | CC | AA | |
| 30 | Male | 60 | Ⅱ | B | Tissue | TT | TT | CG | AG | |
|  |  |  |  |  | Blood | TT | TT | CG | AG | |
| 31 | Female | 56 | Ⅳa | C | Tissue | CT | CT | CG | AA | |
|  |  |  |  |  | Blood | CT | CT | CG | AA | |
| 32 | Male | 65 | Ⅱ | B | Tissue | CT | TT | CC | AA | |
|  |  |  |  |  | Blood | CT | TT | CC | AA | |
| 33 | Male | 52 | Ⅲa | B | Tissue | CT | TT | CC | AA | |
|  |  |  |  |  | Blood | CT | TT | CC | AA | |
| 34 | Male | 70 | Ⅱ | B | Tissue | CT | TT | CG | AA | |
|  |  |  |  |  | Blood | CT | TT | CG | AA | |
| 35 | Male | 50 | Ⅰ | A | Tissue | TT | CT | CC | AA | |
|  |  |  |  |  | Blood | TT | CT | CC | AA | |
| 36 | Male | 70 | Ⅱ | B | Tissue | CT | TT | CC | AA | |
|  |  |  |  |  | Blood | CT | TT | CC | AA | |
| 37 | Male | 55 | Ⅰ | A | Tissue | TT | CT | CG | AG | |
|  |  |  |  |  | Blood | TT | CT | CG | AG | |
| 38 | Male | 57 | Ⅲc | B | Tissue | CT | CT | CC | AA | |
|  |  |  |  |  | Blood | CT | CT | CC | AA | |
| 39 | Male | 62 | Ⅱ | A | Tissue | CT | CT | CG | AG | |
|  |  |  |  |  | Blood | CT | CT | CG | AG | |

The patient with non-matched genotype was shown in bold.

**Additional file 1: Table S6.** Comparative analysis of the clinical characteristics of hepatocellular carcinoma patients with different miR-146a genotypes

| **Characteristic** | **Reference Intervals** | **CC(n=149)** | **CG +GG(n=165)** | ***P*** |
| --- | --- | --- | --- | --- |
| Alanine amiotransferase (U/L) ^a^ | 0-46 | 42.0 (28.5, 82.0) | 36.5 (27.0, 74.3) | 0.391 |
| Aspartate aminotransferase,  (U/L) ^a^ | 0-46 | 47.0 (31.0, 82.0) | 44.5 (30.0, 71.5) | 0.644 |
| Total bilirubin (μmol/L) ^a^ | 0-25 | 19.2 (13.7, 27.7) | 18.2 (13.9, 25.1) | 0.455 |
| Direct bilirubin (μmol/L) ^a^ | 0-7 | 5.2 (3.5, 7.7) | 4.8 (3.6, 6.8) | 0.314 |
| Indirect bilirubin (μmol/L) ^a^ | 1.5-18 | 14.0 (10.0, 19.0) | 14.1 (9.7, 18.5) | 0.818 |
| Total protein (g/L) ^a^ | 60-80 | 68.0 (62.1, 73.9) | 68.4 (62.8, 73.4) | 0.816 |
| Albumin (g/L) ^b^ | 35-55 | 40.5±5.9 | 39.8±6.0 | 0.274 |
| Globulin (g/L) ^a^ | 20-30 | 26.6 (22.4, 30.3) | 27.7 (24.3, 31.2) | 0.097 |
| Albumin / Globulin ^b^ | 1.5-2.5 | **1.6±0.4** | **1.5±0.3** | **0.011** |
| γ-glutamyltransferase (U/L) ^a^ | 5-55 | 59.0 (40.3, 125.8) | 62.5 (38.8, 110.5) | 0.824 |
| Alkaline phosphatase (U/L) ^a^ | 35-134 | 99.0 (75.0, 129.0) | 99.0 (79.0, 132.3) | 0.702 |
| 5-Nucleotidase (U/L) ^a^ | 0-10 | 3.0 (2.0, 7.0) | 3.0 (2.0, 5.0) | 0.499 |
| Total biliary acid (μmol/L) ^a^ | 0-15 | 7.8 (4.0, 19.2) | 6.6 (3.6, 13.2) | 0.186 |
| Cholinesterase (U/L) ^b^ | 3000-10500 | 6145.2±2442.5 | 5947.9±2217.0 | 0.468 |
| Pre-albumin (mg/L) ^b^ | 100-400 | 128.9±65.4 | 122.3±86.7 | 0.470 |
| Glucose (mmol/L) ^a^ | 3.9-6.2 | 5.1 (4.6, 5.8) | 5.0 (4.5, 5.8) | 0.612 |
| Blood urea nitrogen (mmol/L) ^a^ | 1.7-7.2 | 5.1 (3.9, 5.9) | 4.9 (3.6, 6.0) | 0.669 |
| Creatinine (μmol/L) ^a^ | 45-117 | 74.5 (65.5, 83.9) | 73.6 (64.7, 83.9) | 0.679 |
| Uric acid (μmol/L) ^b^ | 119-417 | 261.1±91.3 | 247.4±83.4 | 0.177 |
| Retinol-binding protein (mg/L) ^a^ | 15-70 | 28.2 (16.3, 34.7) | 26.3 (17.9, 35.4) | 0.821 |
| Cystatin C (mg/L) ^a^ | 0-1.2 | 1.0 (0.9, 1.2) | 1.0 (0.9, 1.2) | 0.862 |
| Carcinoembryonic antigen,  (ng/mL) ^a^ | 0-5 | 2.3 (1.5, 3.7) | 2.1 (1.5, 3.4) | 0.538 |
| Alpha-fetoprotein (ng/mL) ^a^ | 0-20 | 227.8 (11.5, 977.5) | 109.1 (8.8, 932.3) | 0.089 |
| Ferritin (ng/mL) ^a^ | 0-322 | 239.5 (152.2, 382.7) | 213.0 (128.0, 431.3) | 0.930 |
| Cancer antigen 125 (KU/L) ^a^ | 0-35 | 14.8 (9.1, 28.0) | 16.4 (9.8, 43.1) | 0.383 |
| Cancer antigen 153 (KU/L) ^a^ | 0-35 | 9.6 (7.1, 12.7) | 10.6 (7.6, 16.3) | 0.122 |
| Cancer antigen 199 (KU/L) ^a^ | 0-35 | 10.0 (4.4, 22.6) | 14.1 (7.5, 28.5) | 0.055 |
| Prothrombin time (sec) ^a^ | 10.5-13.5 | 11.9 (11.1, 12.9) | 12.0 (11.1, 13.1) | 0.932 |
| PT% (%)^b^ | 80-130 | 94.2±21.5 | 93.9±23.5 | 0.923 |
| International standard ratio ^a^ | 0.85-1.15 | 1.0 (1.0, 1.1) | 1.0 (1.0, 1.1) | 0.500 |
| D-fibrinogen (g/L) ^a^ | 2-4 | 2.6 (2.2, 3.2) | 2.9 (2.2, 3.5) | 0.070 |
| activated partial thrombo -plastin time (sec) ^b^ | 28-40 | 34.3±6.2 | 35.0±8.5 | 0.441 |
| Thrombin time (sec) ^a^ | 11-14 | 14.4 (13.8, 15.3) | 14.5 (13.6, 15.7) | 0.510 |

^a^, Data were expressed as median (25th Percentile, 75th Percentile).

^b^, Data were expressed as mean±SD.
